# The prevalence of Duchenne/Becker muscular dystrophy in the Russian Federation

**DOI:** 10.3389/fgene.2026.1953115

**Published:** 2026-10-26

**Authors:** Elena Kistol, Alexander Pushkov, Anton Kiselev, Maria Bulakh, Alena Chukhrova, Peter Sparber, Oksana Ryzhkova, Olga Shchagina, Ilya Zhanin, Marianna Maretina, Kirill Savostyanov, Andrey Glotov, Aleksander Polyakov

**Affiliations:** 1 Research Centre for Medical Genetics, Moscow, Russia; 2 National Medical Research Center for Children’s Health Federal State Autonomous Institution of the Russian Federation Ministry of Health, Moscow, Russia; 3 D.O. Ott Research Institute of Obstetrics, Gynecology and Reproductology, Saint-Petersburg, Russia

**Keywords:** DMD, Duchenne/Becker muscular dystrophy (DMB/BMD), molecular genetic diagnostics, prevalence, selective screening programme

## Abstract

**Introduction:**

Duchenne and Becker muscular dystrophies (DMD/BMD) are among the most common inherited neuromuscular disorders worldwide. The reported prevalence of DMD is 7.1 cases per 100,000 adult males and 2.8 cases per 100,000 individuals in the general population, while the birth prevalence of DMD is estimated at 19.8 cases per 100,000 liveborn males.

**Methods:**

The current study presents comprehensive epidemiological data on DMD/BMD in the Russian Federation. The analyzed cohort comprised 2 776 patients from the Russian Federation with a diagnosis of DMD/BMD confirmed by molecular genetic testing.

**Results:**

According to the obtained results, the estimated birth prevalence of DMD/BMD in the Russian Federation was 20.3 per 100,000 liveborn males (95% CI: 18.8-21.7).

**Discussion:**

The estimated birth prevalence of DMD/BMD in the Russian Federation, together with advances in disease-modifying therapies, particularly gene replacement therapy, supports the need to develop and implement newborn and early childhood screening programs. Such programs could facilitate the initiation of supportive and disease-modifying therapies at early stages, potentially before the onset of clinical symptoms, thereby improving disease outcomes and prognosis. The results of the present study are also important for estimating future treatment needs and planning healthcare expenditures.

## 1 Introduction

Duchenne/Becker muscular dystrophy (DMD/BMD) is one of the most severe forms of inherited muscular dystrophies and one of the most common hereditary neuromuscular disorders worldwide. DMD was first described in detail and clinically characterized in 1830, when its principal symptoms were identified (Huml, 2015). Much later, in 1955, an allelic form of DMD - Becker muscular dystrophy (BMD) - was described; it is characterized by a similar clinical phenotype but a milder course and later onset (Gaynetdinova, 2020).

For a prolonged period following the initial description of the disease, the causative gene remained unknown. The *DMD* gene was identified on the X chromosome only in 1986, and the dystrophin protein was subsequently characterized, which allowed to determine the molecular mechanisms underlying the pathogenesis of DMD/BMD (Koenig et al., 1987).

The first attempts to determine the prevalence of DMD/BMD were undertaken in the 1970 s in southwestern Germany, in the city of Freiburg, as part of a newborn screening program (1974). In this program, elevated creatine kinase (CK) levels were detected in 5 of 8,590 newborn boys, and DMD was subsequently clinically confirmed. However, this data was considered overestimated, as participation in the program was voluntary and predominantly involved families with a burdened anamnesis. Subsequently, over the course of the program from 1977 to 2011, the incidence of DMD was established as 1:3,600 liveborn boys, and that of BMD as 1:15,500 liveborn boys (Scheuerbrandt, 2018). In 1991, similar incidence rates for DMD/BMD (DMD - 1:3,500; BMD - 1:17,800) were reported based on a comprehensive review of the global literature encompassing more than 150 published studies (Emery, 1991). Nevertheless, despite this data, newborn screening for DMD was not widely implemented in clinical practice, as effective molecular genetic diagnostics and therapy were not available for patients with this disease.

At present, DMD/BMD is considered the most common and severe inherited neuromuscular disorder worldwide. The overall prevalence of DMD is estimated at 7.1 cases per 100,000 among the adult male population and 2.8 cases per 100,000 in the general population, while the incidence of DMD among liveborn boys is 19.8 cases per 100,000. The average prevalence of BMD is estimated to be 1.53–1.6 cases per 100,000 individuals (Crisafulli et al., 2020). However, comprehensive data on the prevalence of DMD/BMD in the Russian Federation has not previously been reported.

As of date, advances in knowledge regarding disease etiology, the structure of the dystrophin gene, characteristics of the spectrum of pathogenic and likely pathogenic variants, as well as the development of molecular genetic diagnostic methods, have led to the accumulation of data sufficient to estimate the prevalence of DMD/BMD in the Russian Federation.

Determining the prevalence of DMD/BMD primarily provides information on the genetic burden, population structure, and degree of genetic load. In addition, epidemiological data makes it possible to estimate healthcare expenses, including the number of medical staff members required for patient management, costs of molecular genetic diagnostics and medical services, and the number of medications needed for supportive and symptomatic therapy. Assessment of DMD/BMD prevalence also allows to determine the proportion of patients eligible for modern etiopathogenetic therapies and to calculate the required level of funding.

The determination of DMD/BMD prevalence also raises the question of implementing neonatal or early selective screening in the Russian Federation, as three approaches to etiopathogenetic therapy are currently available, including gene replacement therapy, which in the near future will become accessible to a broad group of patients regardless of the type of nucleotide sequence variant.

## 2 Materials and methods

### 2.1 Study sample

The results of a selective screening program for DMD/BMD from 2018 to 2025 conducted at three federal centers of the Russian Federation were analyzed: the Federal State Budgetary Scientific Institution “N. P. Bochkov Research Centre for Medical Genetics” (Moscow), the Federal State Autonomous Institution “National Medical Research Center for Children’s Health” of the Ministry of Health of the Russian Federation (Moscow), and the Federal State Budgetary Scientific Institution “D. O. Ott Research Institute of Obstetrics, Gynecology and Reproductology” (Saint Petersburg).

Biological samples were collected in laboratory units of medical genetic counseling centers across various regions of the Russian Federation. Written informed consent for participation in the study was obtained from all patients, their parents or legal guardians (in case of underage patients).

The main inclusion criteria for the study were: (1) male sex, age ≤29 years; (2) citizenship of the Russian Federation; (3) a serum creatine kinase (CK) level of at least 1000 U/L as determined by biochemical testing and/or a clinically established diagnosis of DMD/BMD. The data was compared and cross-checked to identify duplicate patients across centers, as some individuals were referred for retesting. For the analysis of DMD/BMD prevalence, each patient was counted only once. The analyzed cohort comprised 2,776 patients from the Russian Federation with a diagnosis of DMD/BMD confirmed by molecular genetic methods.

### 2.2 Molecular genetic diagnostic

Within the selective screening program, the first stage of screening was the search for gross deletions and duplications of one or several exons of the DMD gene by means of multiplex ligase-dependent probe amplification (MLPA) using commercially available kits (MLPA SALSA P034 and P035 DMD probemix, MRC-Holland, Netherlands). The reaction product was detected with fragmentary analysis on ABI Prism 3,500 (“Applied Biosystems”). The data were analyzed using the Coffalyser. Net™ software.

The second stage of diagnostics was a search for point variants using a targeted gene panels the panel used at the Research Centre for Medical Genetics included the following genes: *DMD, CAPN3, EMD, SGCG, SGCA, SGCB, SGCD, TCAP, FKRP, POMT1, POMT2, ANO5, FKTN, ISPD, LMNA, CAV3, DAG1, POPDC3, FHL1, GAA, PLEC, POMGNT1, POMGNT2, GMPPB, HNRNPDL, GNE, FKBP14, DYSF, DNAJB6, BVES, TRIM32*; at the National Medical Research Center for Children’s Health of the Ministry of Health of the Russian Federation, an expanded targeted panel including 248 genes was used), whole-exome sequencing.

Analysis of the dystrophin gene coding sequence using these methods yields a high detection rate for pathogenic and likely pathogenic DMD variants, confirming the diagnosis in the overwhelming majority of cases. However, despite this, cases of DMD/BMD may remain undiagnosed, especially in patients with deep intronic variants and complex structural rearrangements.

In cases where genetic testing fails to confirm a clinical diagnosis of DMD, muscle biopsy specimens should be evaluated for dystrophin protein expression via immunohistochemistry or Western blotting. A significant proportion of mutations have also been shown to affect RNA expression and/or processing, and therefore RNA sequencing may serve as an important diagnostic tool for patients with DMD/BMD without confirmed molecular genetic analysis.

### 2.3 Prevalence estimate

The estimation of the prevalence of DMD/BMD included an assessment of the overall incidence of DMD, which was calculated as the ratio of the number of DMD cases registered and molecularly confirmed at three federal centers of the Russian Federation to the total number of liveborn male in the country according to data from the Federal State Statistics Service (Rosstat) for the corresponding calendar year (Central Research Institute of Healthcare Organization and Informatization, 127254, Moscow, Russia, 2024) (Equation 1). The frequency of DMD was calculated for children stratified by year of birth.

$$f=\frac{n}{N}$$ (1)

where f is the disease frequency;

n is the absolute number of children with DMD/BMD molecularly confirmed within a given calendar year; N is the number of liveborn males in the Russian Federation according to the Federal State Statistics Service (Rosstat) in the same calendar year.

### 2.4 Statistical analysis

Statistical data analysis was performed using the Statistica 10.0 software package. The 95% confidence interval (95% CI) was calculated using the Clopper–Pearson method.

For statistical analysis, the standard chi-square (χ^2^) test was used to compare the frequency distribution of a single characteristic between the two samples. Values ​​with a significance level of p < 0.05 were considered statistically significant for all tests used.

## 3 Results

Molecular genetic diagnostics of patients with Duchenne/Becker muscular dystrophy within selective screening programs was performed at three federal centers of the Russian Federation from 2018 to 2025: N. P. Bochkov Research Centre for Medical Genetics (Moscow), the National Medical Research Center for Children’s Health of the Ministry of Health of the Russian Federation (Moscow), and the D. O. Ott Research Institute of Obstetrics, Gynecology and Reproductology (Saint Petersburg).

The program included patients from 80 regions of the Russian Federation. The prevalence of DMD/BMD was assessed cumulatively for all patients across the Russian Federation, since within the selective screening program it was often difficult to determine the patient’s region of birth. This was due to incomplete questionnaire data and limited available information, as well as the absence of medical genetic centers in some cities and the follow-up of patients in reference and interregional centers of the Russian Federation.

Since 2018, the three federal centers have accumulated extensive data on the results of DNA diagnostics of Russian patients with DMD/BMD. At the time of referral for genetic counseling, the patients’ ages ranged from 7 months to 29 years (median 7 years 7 months). The data was compared and cross-checked to identify duplicate patients across centers, as some individuals were referred for retesting. For the analysis of DMD/BMD prevalence, each patient was counted only once. During the specified period, pathogenic and likely pathogenic variants in the *DMD* gene were identified in 2,776 patients. On the basis of this data, an analysis of DMD/BMD prevalence in the Russian Federation was performed. To determine disease prevalence, patients with pathogenic and likely pathogenic variants in the *DMD* gene (n = 2,776) were stratified by year of birth. The patient birth year ranged between 2000 and 2024 (Figure 1).

**FIGURE 1:**
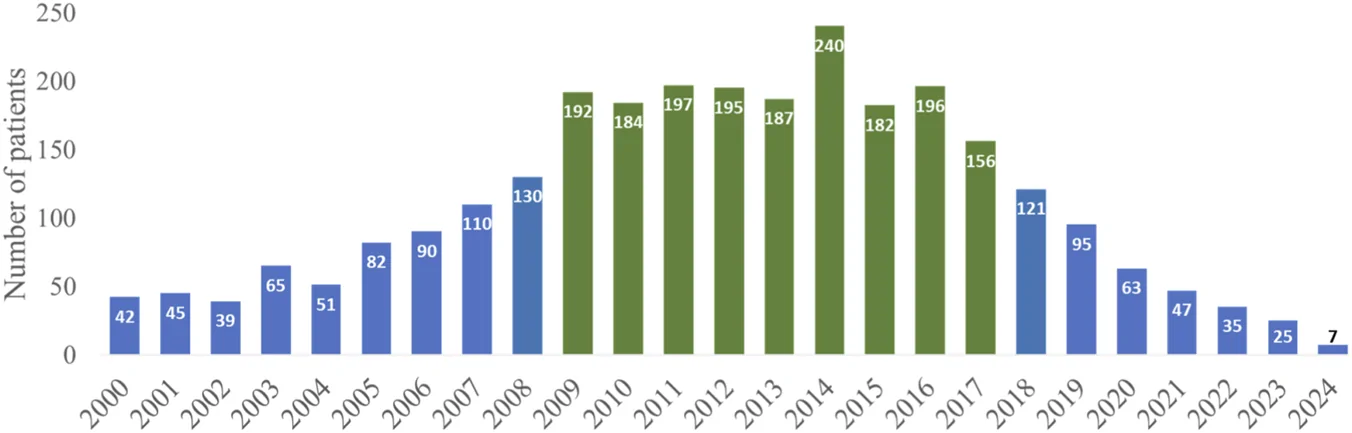
Patients with a confirmed diagnosis of Duchenne/Becker muscular dystrophy (green indicates the cohort of patients with the assessed prevalence of DMD/BMD born between 2009 and 2017).

The prevalence of DMD/BMD among children was calculated according to patients’ year of birth. The overall incidence of DMD/BMD was estimated as the ratio of the number of DMD/BMD cases with a molecularly confirmed diagnosis to the total number of liveborn males in the country according to data from the Federal State Statistics Service (Rosstat) for the corresponding calendar year. The obtained data is presented in Table 1.

**TABLE 1:** Number of live births in the Russian Federation and the number of patients with DMD/BMD born between 2000 and 2023.

Year of birth	Number of patients with DMD/BMD	Number of births (according to Rosstat)	DMD/BMD prevalence among all newborns	DMD/BMD prevalence among all liveborn males
2000	42	1 266 800	3.3:100,000	6.6:100,000
2001	45	1 311 604	3.4:100,000	6.9:100,000
2002	39	1 396 967	2.8:100,000	5.6:100,000
2003	65	1 477 301	4.4:100,000	8.8:100,000
2004	51	1 502 477	3.4:100,000	6.8:100,000
2005	82	1 457 376	5.6:100,000	11.3:100,000
2006	90	1 479 637	6.1:100,000	12.2:100,000
2007	110	1 610 122	6.8:100,000	13.7:100,000
2008	130	1 713 947	7.6:100,000	15.2:100,000
**2009**	**192**	**1 761 687**	**10.9:100,000**	**21.8:100,000**
**2010**	**184**	**1 788 948**	**10.3:100,000**	**20.6:100,000**
**2011**	**197**	**1 796 629**	**11.0:100,000**	**21.9:100,000**
**2012**	**195**	**1 902 084**	**10.3:100,000**	**20.5:100,000**
**2013**	**187**	**1 895 822**	**9.9:100,000**	**19.7:100,000**
**2014**	**240**	**1 942 683**	**12.4:100,000**	**24.7:100,000**
**2015**	**182**	**1 940 579**	**9.4:100,000**	**18.8:100,000**
**2016**	**196**	**1 888 729**	**10.4:100,000**	**20.8:100,000**
**2017**	**156**	**1 690 307**	**9.2:100,000**	**18.5:100,000**
2018	121	1 604 344	7.5:100,000	15.1:100,000
2019	95	1 481 074	6.4:100,000	12.8:100,000
2020	63	1 435 800	4.4:100,000	8.8:100,000
2021	47	1 402 800	3.4:100,000	6.7:100,000
2022	35	1 306 200	2.7:100,000	5.4:100,000
2023	25	1 265 000	2.0:100,000	4.0:100,000

Patient groups for which the prevalence of DMD/BMD, in the Russian Federation was estimated are shown in bold.

According to the obtained data, it is more appropriate to assess the prevalence of DMD/BMD in the group of patients born between 2009 and 2017 (n = 1,729). This is because the number of patients with DMD/BMD born in 2000–2008 and 2018–2019 in the analyzed cohort was significantly lower (Figure 1). However, the decrease in the number of patients in these periods is not associated with changes in birth rates, as confirmed by data from the Federal State Statistics Service (Rosstat) (Table 1). In addition, when calculating the prevalence of DMD/BMD by year of birth, a peak increase in incidence was observed from 2009 to 2017 (18.8–24.7 per 100,000). This can be explained by the fact that patients born before 2009 were much less frequently referred for molecular genetic testing because of limited methodological capabilities of diagnostic laboratories at that time, as well as the absence of free selective screening programs. Thus, patients of older age groups (born before 2009) are likely underrepresented in selective screening programs, and their numbers are insufficient to reliably estimate the disease prevalence. Furthermore, patients born after 2017 are not yet fully covered by molecular genetic testing, as the average age of clinical manifestation of DMD/BMD is 4–6 years. We hypothesize that these patients remain underdiagnosed and may often be missed in routine clinical practice, as the median age at diagnosis, according to our data, is 7 years 7 months.

According to data provided by Rosstat, 16,607,468 children were born between 2009 and 2017 (https://rosinfostat.ru/rozhdaemost/). Among them, 1,729 cases of DMD/BMD were identified. Based on this data, the average prevalence of DMD/BMD was estimated to be 10.4 per 100,000 (95% CI: 9.7–11.1) among all live births. Taking into account that there are 105 male births per 100 female births (Chao et al., 2019), the disease prevalence is 20.3 per 100,000 (95% CI: 18.8–21.7) liveborn males (Table 1).

According to various estimates, the global incidence of DMD/BMD is 19.8 per 100,000 liveborn males (95% CI: 16.6–23.6). This data was obtained from an analysis of 40 publications on the prevalence of the disease among children and newborns (Salari et al., 2022). In the Russian Federation, the prevalence of DMD/BMD was 20.3 per 100,000, which is comparable to the global estimates reported by Crisafulli S. et al. (2020).

We also examined the spectrum of pathogenic and likely pathogenic variants in the DMD gene among patients with a molecularly confirmed diagnosis of DMD/BMD, for whom relevant genetic information was accessible (2455 probands). The distribution of variants types in Russian DMD/BMD patients is shown in Figure 2. Gross deletions and duplications were detected in 58.1% (1426/2455) and 14.0% (343/2455) of patients, respectively (Figure 2A).

**FIGURE 2:**
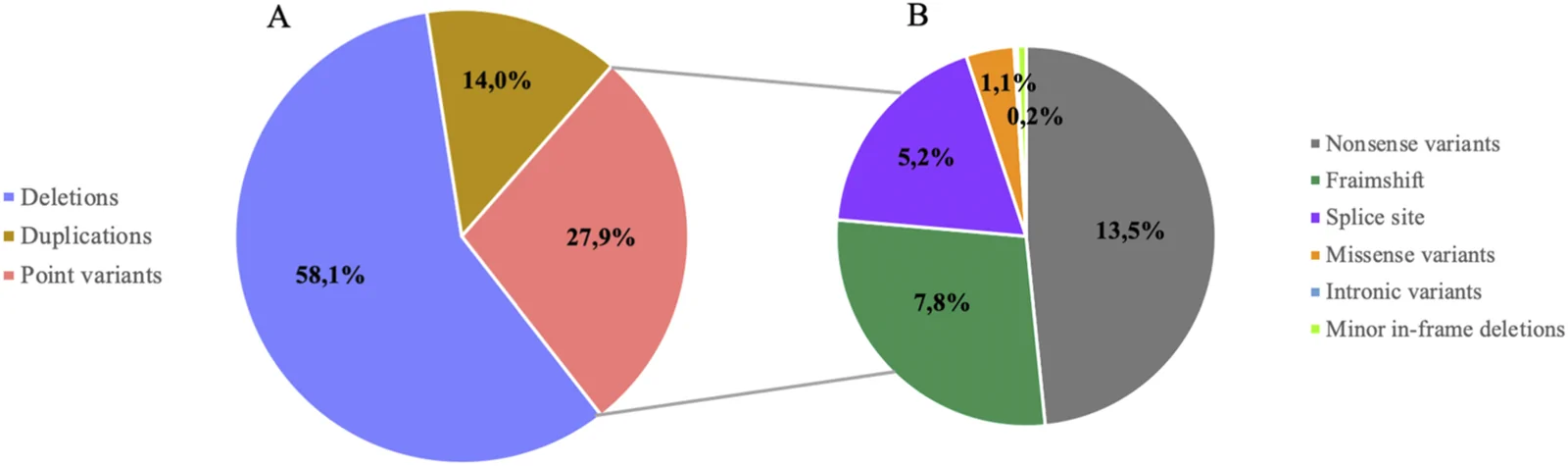
**(A)** Spectrum of pathogenic and likely pathogenic variants in the DMD gene in a Russian cohort of patients with DMD/BMD. **(B)** Spectrum of point variants in the DMD gene.

Point variants comprised approximately 27,9% (686/2455) of all pathogenic and likely pathogenic variants in our cohort. It is worth noting that among point variants, the most frequent ones were nonsense variants – 13.5% (332/2455). Aside from that, we detected minor deletions/duplications/insertions, leading to a frameshift (192/2455, 7.8%), splice site variants (127/2455, 5.2%), missense variants (28/2455, 1.1%), and deep intronic variants (2/2455, 0.08%) and minor in-frame deletions – 0.2% (5/2455) each (Figure 2B). These expanded data are in agreement with those from an earlier study examining the mutational spectrum in Russian patients (Zinina, 2022).

## 4 Discussion

At present, several approaches are used to estimate disease prevalence in the population: (1) implementation of universal neonatal screening; (2) assessment based on carrier frequency; and (3) selective screening of patients. One of the most accurate and effective approaches is estimation based on the results of mass neonatal screening. However, to date, neonatal screening programs for DMD/BMD have not been implemented anywhere in the world. This is due to the lack of a standardized algorithm for testing the muscle isoform of creatine kinase (CK) and for subsequent molecular genetic diagnostic pathways in patients with elevated enzyme levels. In addition, there is currently no universal treatment for DMD/BMD, unlike, for example, spinal muscular atrophy, suitable for all patients regardless of the mutation type. The majority of currently available treatment strategies for DMD are mutation-specific and include approaches such as exon skipping and therapies targeting premature termination codons.

Nevertheless, with the recent emergence of effective symptomatic and etiopathogenetic mutation-specific treatment for DMD/BMD (particularly gene replacement therapy) and the increasing proportion of patients for whom such approaches are available, the issue of organizing and implementing neonatal screening has become especially relevant (Kemper et al., 2025). Thus, in December 2025, the U.S. Department of Health and Human Services approved the inclusion of DMD in the list of conditions recommended for mass neonatal screening (Kemper et al., 2025).

Despite the aforementioned difficulties, pilot neonatal screening programs for DMD/BMD have been conducted and are now widely implemented. Based on their results, it has become possible to estimate the prevalence of the disease globally, as well as in specific countries. Since 1975, approximately two million newborns have been screened in 15 medical centers worldwide within pilot programs. As a result, markedly elevated creatine kinase levels were detected in 363 individuals; as a result, a clinical diagnosis of DMD/BMD was confirmed. This aggregated data made it possible to estimate a disease prevalence of 1:5,509 liveborn males (Ji et al., 2024).

However, these results should be considered unreliable, seeing as in most cases molecular genetic diagnostics were not performed for patients with elevated creatine kinase levels, primarily due to methodological limitations of laboratories during the early stages of the pilot projects. In addition, within pilot screening programs, neonatal samples are typically collected within the first 24–72 h of life, when elevated levels of the muscle isoform of creatine kinase may also result from traumatic birth-related events, leading to a high number of false-positive results (Potter et al., 2024). It has also been shown that creatine kinase levels increase significantly at 4–6 weeks of age in patients with DMD/BMD; therefore, screening for DMD/BMD after 2 months of age may be more appropriate in order to reduce diagnostic errors and avoid missing patients (Mendell et al., 2012).

Previously, researchers in the United States reported the results of a pilot screening study in which 16 of 15,754 newborns (1 in 985) were identified with creatine kinase (CK) levels exceeding threshold values that varied depending on the age of the newborn at the time of blood sampling. The normal reference range was defined as CK < 1,990 ng/mL when blood was collected within 0–47 h of life, and <571 ng/mL when collected within 72–167 h of life. When threshold values were exceeded, the newborn was referred to a specialist to determine further diagnostic and clinical management (Park et al., 2022).

Thus, pilot programs of selective and neonatal screening emphasized the importance and advantages of early diagnosis of DMD/BMD, not only for determining disease prevalence but also for ensuring timely access to gene replacement therapy at an early age. At present, most medications for the treatment of DMD have been approved and clinically tested primarily in patients older than 2 years of age. Consequently, the applicability of etiopathogenetic therapy in patients identified through neonatal screening at approximately 2 months of age remains uncertain. The potential for expanding age criteria for therapy initiation is supported by published data demonstrating the long-term safety and efficacy of gene replacement therapy in children younger than 3 years of age (Chwalenia et al., 2025).

Another method for estimating disease prevalence is calculation based on carrier frequency. However, in the case of DMD/BMD, determination of carrier frequency is relatively labor-intensive, as elevated serum creatine kinase levels are observed in only 50%–70% of heterozygous female carriers, and some asymptomatic carriers may therefore be missed (Han et al., 2020). In addition, determining carrier frequency through molecular genetic testing of women is financially costly due to the wide spectrum of pathogenic and likely pathogenic variants in the *DMD* gene and the absence of common nucleotide variants that could serve as a first-line diagnostic target. Furthermore, estimation of DMD/BMD prevalence based on carrier rates is complicated by the fact that approximately 30% of dystrophin gene variants arise *de novo* (Sakthivel Murugan et al., 2013), and cases of germline mosaicism have been reported in 3.6% of families (Verebi et al., 2025).

At present, the optimal approach for determining the prevalence of DMD/BMD in the Russian Federation is the analysis of selective screening programs. Diagnostic evaluation for DMD/BMD in Russian patients has been conducted for a prolonged period, beginning in the late 1980 s. Each year, the informativeness of diagnostics has steadily increased, primarily due to greater awareness and vigilance among physicians and parents, as well as the emergence of patient organizations and foundations. In addition, methodological approaches to molecular genetic diagnostics have expanded and evolved, currently enabling the detection of rare *DMD* gene variants, such as deep intronic variants and complex structural rearrangements. Furthermore, with the emergence of promising etiopathogenetic therapeutic approaches in 2018, selective screening programs were widely implemented in three centers in the Russian Federation: N. P. Bochkov Research Centre for Medical Genetics (Moscow), the National Medical Research Center for Children’s Health (Moscow), and the D. O. Ott Research Institute of Obstetrics, Gynecology, and Reproductology (Saint Petersburg).

Owing to the implementation and expansion of selective screening programs, it has been possible to estimate the prevalence of DMD/BMD in the Russian Federation for the first time as 10.4 per 100,000 (95% CI: 9.7–11.1) among all newborns and 20.3 per 100,000 (95% CI: 18.8–21.7) among male newborns. A systematic analysis of DMD/BMD prevalence has shown that reported rates vary substantially across studies depending on ethnic background, and differences in prevalence are observed even between regions within the same country. For example, in Taiwan the prevalence of DMD/BMD is 11.5 per 100,000 liveborn males, whereas in China the reported prevalence is 25.6 per 100,000 and 22.8 per 100,000 liveborn males in Hangzhou and Henan provinces, respectively (Jia et al., 2023). All this data was obtained from pilot neonatal screening programs.

Table 2 presents the prevalence of the disease in different countries worldwide. For comparison, cohorts were selected in which the prevalence of DMD/BMD was assessed in children and in newborns.

**TABLE 2:** Cumulative estimates of DMD and BMD prevalence in different countries.

Country (years of study)	Patient age	Number of patients with DMD/BMD	Number of liveborn males during the study period	DMD/BMD prevalence among liveborn males	DMD/BMD prevalence per 100,000 liveborn males
United States (1982–2011) (Romitti et al., 2015)	5–24	649	3,470,128	1:5,347	18.7:100,000
United Kingdom (Wales)* (1990–2011) (Moat et al., 2013)	0	72	369,780	1:5,136	19.5:100,000
Finland (1992–2018) (Muuronen et al., 2025)	0–17	17	100,284	1:5,899	16.8:100,000***
Canada (2000–2009) (Mah et al., 2011)	0–24	773	5,000,555	1:6,469	15.5:100,000***
Australia (2002–2012) (Kariyawasam et al., 2022)	10–20	107	541,899	1:5,076	19.7:100,000
Norway (2005) (Rasmussen et al., 2015)	0–18	40	197,922	1:4,948	20.2:100,000
China (Henan province) (2022)* (Jia et al., 2023)	0	3	13,110	1:4,370	22.8:100,000
Russian Federation** (2018–2024)	0–24	1729	16,607,468	1:4,920	20.3:100,000

* Incidence data based on pilot neonatal screening programs.

** Current study.

*** significant values with p < 0.05 (Chi-squared test).

Based on the analyzed data, it can be concluded that epidemiological estimates of DMD/BMD prevalence are comparable across most studies. For example, the incidence observed in Russian patients is very close to that reported in the United Kingdom and Australia and is almost identical to that reported for Norway. However, some differences are observed when compared with Finland and Canada, where the incidence of DMD/BMD is 16.8 and 15.5 cases per 100,000 male newborns, respectively, whereas in the present study it is 20.3 per 100,000 (Table 2). Differences are also noted in comparison with data reported by Chinese researchers, where the incidence is 22.8 per 100,000. It is assumed that these differences in incidence are related to study design, since, for example, in Finland the diagnostic approach targeted the most common hereditary neuromuscular diseases, and a separate selective screening program for DMD/BMD was not implemented (Muuronen et al., 2025).

Variability in the reported frequency of DMD/BMD is observed not only between individual countries but also across different combined studies. According to previously published summary and combined studies, the prevalence of DMD/BMD ranges from 0.9 to 16.8 cases per 100,000 males across 15 studies published between 1982 and 2016, and from 0.7 to 7.7 cases per 100,000 individuals in the general population across five studies conducted between 1977 and 2005 (Lorenz et al., 2025). Such variability is primarily attributable to differences in sample size and in the methods used for molecular genetic diagnosis and population-based analyses. In addition, the lack of available data from many countries prevents a comprehensive assessment of disease prevalence in children, and according to aggregated data, prevalence estimates remain heterogeneous, with exact values still unknown (Mah et al., 2014).

Previously, attempts to estimate the prevalence of DMD/BMD in the Russian Federation were undertaken; however, these efforts were limited to individual regions, and no unified data was available. In the Rostov Oblast, the prevalence of the disease among the newborn males was reported as 21.6 per 100,000 (1:4,629) (Research Centre of Medical Genetics of the Russian Academy of Sciences, 2019). In the Republic of North Ossetia–Alania, the prevalence of DMD/BMD was estimated at 1 in 3,912 male newborns (95% CI: 1:2,584–1:6,242), corresponding to 25.5 per 100,000, which is higher than that observed in the present study and in previously published studies worldwide (Zinchenko et al., 1458). Furthermore, the prevalence data for DMD/BMD obtained in this study may be underestimated, as it is impossible to fully assess the diagnostic coverage. It was not possible to determine the incidence of the disease in different regions of the Russian Federation, and the analysis was conducted based on a specific time frame (birth years 2009–2017).

Thus, analysis of disease prevalence in relation to birth rates is of considerable importance for estimating the number of identified DMD/BMD cases, particularly given the availability of modern etiopathogenetic therapeutic approaches that continue to be actively developed and implemented in clinical practice. Complete results of selective screening in the Russian Federation are aggregated into the national registry of DMD/BMD (Sparber et al., 2025).

## 5 Conclusion

Thus, the performed prevalence analysis indicates that DMD/BMD is one of the most common hereditary neuromuscular diseases in the Russian Federation. In addition, the estimation of disease incidence in the Russian Federation, together with advances in etiopathogenetic therapies - particularly gene replacement therapy, which is expected to become available to a larger proportion of patients regardless of the specific nucleotide variant -supports the need to develop and implement neonatal and early childhood screening programs. Such programs would enable the initiation of symptomatic and pathogenetic therapy at early stages, prior to the onset of symptoms, thereby improving disease course and prognosis. The results of the present study are also important for the prediction of therapeutic requirement and for planning medical budgets.

## Data Availability

The original contributions presented in the study are included in the article/supplementary material, further inquiries can be directed to the corresponding author.
